# Clonal Hematopoiesis of Indeterminate Potential as an Emerging Interdisciplinary Risk Factor in Alzheimer’s Disease: Current Evidence and Future Directions

**DOI:** 10.3390/biomedicines14051012

**Published:** 2026-04-29

**Authors:** Klara Kopp, Patricia Silva, Frederik Damm, Nicoleta Carmen Cosma

**Affiliations:** 1 Department of Hematology and Tumor Immunity, Charité—Universitätsmedizin Berlin, Augustenburger Platz 1, 13353 Berlin, Germany; klara.kopp@charite.de (K.K.); patricia-alexandra.santos-silva@charite.de (P.S.); frederik.damm@charite.de (F.D.); 2 Department of Psychiatry and Neurosciences, Charité—Universitätsmedizin Berlin, Hindenburgdamm 30, 12203 Berlin, Germany; 3 BIH Charité Clinician Scientist Program, BIH Biomedical Innovation Academy, Berlin Institute of Health, Charité—Universitätsmedizin Berlin, Anna-Louisa-Karsch-Str. 2, 10178 Berlin, Germany

**Keywords:** clonal hematopoiesis of indeterminate potential (CHIP), Alzheimer’s disease, neuroinflammation, vascular dementia, somatic mutations, mixed dementia, brain aging, survivor bias

## Abstract

Clonal hematopoiesis of indeterminate potential (CHIP) is an age-related condition affecting over 10–20% of individuals older than 70 years, characterized by the expansion of hematopoietic stem cell clones carrying somatic mutations in leukemia-associated driver genes in the absence of overt hematologic disease. Initially recognized as a precursor to hematologic malignancies, CHIP has since been implicated in diverse non-malignant disorders, notably increasing the risk of cardiovascular events by 40%. Recent epidemiological and experimental evidence suggests a potential disease-modifying influence of CHIP in neurodegenerative diseases, particularly Alzheimer’s disease (AD), although findings remain heterogeneous and sometimes contradictory. This review synthesizes recent evidence linking CHIP to AD risk, neuropathology, and disease progression. In this study, we summarize population-based cohort studies reporting a 36 to 54% reduction in the odds of clinical AD among CHIP carriers, alongside emerging data indicating that *DNMT3A* and *TET2* mutations may exert divergent effects on neurodegeneration. Mechanistic insights from experimental models are examined, highlighting the ability of mutated myeloid cells to infiltrate the central nervous system and modulate neuroinflammation and amyloid clearance. We discuss conflicting findings and analyze how CHIP-driven vascular disease and stroke confound neuroprotective signals. We propose that CHIP may differentially influence AD and vascular contributions to cognitive impairment and dementia, shaping mixed dementia phenotypes. Methodological challenges, including survivor bias, competing risks, variable mutation detection thresholds, and incomplete Apolipoprotein E stratification, are discussed. Ultimately, our review clarifies that CHIP is not a simple protective factor, but a complex systemic modulator that reshapes the neurodegenerative and vascular drivers of cognitive decline, necessitating cross-disciplinary neuro-hematology collaboration to establish its role as a novel risk stratificator for improving diagnostic precision and personalizing clinical outcomes in Alzheimer’s disease.

## 1. Introduction

Aging is accompanied by the progressive accumulation of somatic genetic alterations across multiple tissues, creating cellular mosaicism that influences complex organ function. In the hematopoietic system, this phenomenon is termed clonal hematopoiesis of indeterminate potential (CHIP), a condition defined by the expansion of blood cell clones carrying somatic mutations in genes recurrently altered in hematologic malignancies [1]. Although CHIP is typically asymptomatic and does not meet the criteria for a hematologic disorder, its prevalence increases markedly with age, reaching approximately 39% in individuals older than 60 years when utilizing high-sensitivity sequencing (Variant Allele Fraction (VAF) greater than 0.5%) [2,3]. Over the past decade, CHIP has emerged as a clinically relevant state associated with increased all-cause mortality and a broad spectrum of non-malignant diseases, most prominently atherosclerotic cardiovascular disease and stroke [1,4,5].

The recognition that CHIP contributes to systemic inflammation and vascular pathology has reshaped traditional views of age-related disease risk. Seminal experimental and epidemiological studies have demonstrated that CHIP-associated mutations in myeloid cells promote inflammatory signaling and accelerate atherosclerosis, establishing a causal link between somatic mosaicism and chronic disease and providing a biological bridge to neurodegeneration [6]. Given the central role of inflammation, vascular integrity, and immune–brain interactions in neurodegeneration, interest has increasingly turned to the potential relevance of CHIP in disorders of the aging brain [7].

Alzheimer’s disease (AD), the most common cause of dementia worldwide, is now biologically defined by the A/T/N (Amyloid, Tau, and Neurodegeneration) classification rather than clinical symptoms alone [8]. While traditionally conceptualized as a primary neurodegenerative disorder driven by amyloid-beta and tau, AD is increasingly viewed from a geroscience perspective as a multifactorial condition shaped by systemic aging processes, including immune dysregulation, vascular disease, and metabolic dysfunction [9]. Large-scale epidemiological studies have identified substantial overlap between AD and vascular risk factors, and mixed dementia phenotypes are now understood to be the rule rather than the exception in older populations [9]. Within this context, CHIP represents a biologically plausible modifier of brain aging, with the potential to influence neurodegenerative trajectories through both immune-mediated and vascular mechanisms.

Recent studies have reported associations between CHIP and Alzheimer’s disease that are both intriguing and seemingly contradictory. On one hand, evidence for protective effects stems from large-scale population-based analyses, encompassing cohorts ranging from approximately 5000 to over 500,000 individuals, suggest that CHIP carriers may exhibit a significantly reduced incidence of clinically diagnosed AD [10,11]. Conversely, smaller, biomarker-focused cohorts (*n* < 100) and specific genotype-stratified sequencing projects (*n* ≈ 30,000) indicate a different trajectory, reporting either neutral effects or an increased risk of AD pathology [12,13]. Experimental work in mouse models (such as 5xFAD and APP/PS1), utilizing bone marrow chimeric and parabiosis systems, has further added complexity, demonstrating that CHIP-derived myeloid cells can infiltrate the central nervous system and modulate microglial function and amyloid clearance in these mice [14]. Together, these findings challenge simplistic interpretations and underscore the need for a nuanced, integrative appraisal of the CHIP-AD relationship [7].

A critical yet underappreciated aspect of this emerging literature is the strong and well-established association of CHIP with vascular disease, stroke, and cerebrovascular pathology [15,16]. Given that vascular contributions to cognitive impairment and dementia (VCID) frequently coexist with Alzheimer’s pathology, the possibility that CHIP differentially influences neurodegenerative and vascular processes warrants careful consideration [17]. Apparent protective associations with AD may, in part, reflect competing risks, recruiting bias, or survivor bias in populations with elevated vascular morbidity [18,19]. Disentangling these effects is essential for interpreting existing data and designing future studies that accurately capture the complexity of late-life cognitive decline.

In this comprehensive review, we synthesize recent evidence published between 2023 and 2025 examining the relationship between clonal hematopoiesis and Alzheimer’s disease. We integrate epidemiological, experimental, and mechanistic findings, critically assess methodological challenges and sources of heterogeneity, and discuss the confounding role of vascular disease and mixed dementia phenotypes. Finally, we highlight the urgent need for interdisciplinary collaboration among hematology, neurology, and psychiatry and outline strategies for incorporating CHIP assessment into Alzheimer’s cohorts and clinical trials. This framework conceptualizes CHIP as a dynamic systemic modifier, shifting the focus from simple risk association toward an integrative understanding of aging brains.

## 2. Pathophysiology of Alzheimer’s Disease and Immune Involvement

AD accounts approximately 60–70% of dementia cases worldwide and represents a major public health burden [20]. Pathological processes underlying AD begin decades before clinical symptoms emerge, indicating the involvement of long-term interactions between aging, genetic predisposition, and systemic drivers [21]. AD is neuropathologically characterized by the accumulation of extracellular amyloid-ß plaques and intracellular tau tangles, leading to synaptic dysfunction and progressive neuronal loss [22]. Established risk factors for AD include advanced age > 65 years, female sex, and most importantly, the Apolipoprotein E (ApoE) ε4 allele [20,23]. While ApoE ε4 eleveates risk of AD **by about three–four-fold**, ApoE ε2 is associated with a protective effect, and ApoE ε3, as the most prevalent isoform in the general population, is considered largely neutral [23].

Genome-wide association studies have consistently implicated immune-related pathways in AD susceptibility, particularly those involving microglial function [21,22]. Beyond the central nervous system, systemic inflammation and peripheral immune alterations have been linked to cognitive decline and neurodegeneration, suggesting a dynamic neuroimmune crosstalk where peripheral hematopoietic signals propagate to the CNS to drive neurodegeneration. This neuroimmune crosstalk is further supported by recent findings identifying specific epigenetic signatures in peripheral myeloid cells that correlate with neurovascular aging and the accelerated accumulation of somatic mutations within brain-resident immune niches [24,25]. When activated, microglia, the resident immune cells of the central nervous system, play a central role in amyloid clearance, synaptic remodeling, and the regulation of inflammatory signaling within the brain [21]. While parenchymal microglia are primarily maintained independently of adult hematopoiesis, there is growing evidence that age-related dysfunction takes place in neurovascular units, suggesting that during neurodegeneration, the integrity of the blood–brain barrier declines. This may facilitate debated levels of peripheral monocytes to infiltrate the central nervous system and differentiate into microglia-like cells. Within this context, any somatic mutations present in the peripheral hematopoietic system could theoretically be imported into the brain’s immune environment, posing a mechanistic link between systemic aging and local neuroinflammation [10,24,25,26].

## 3. Clonal Hematopoiesis: Definition, Prevalence, and Biological Consequences

Functionally, clonal hematopoiesis-associated mutations alter the behavior of hematopoietic stem cells (HSCs); some also confer a fitness advantage, leading to clonal expansion [27,28]. This expansion is characterized by a distinct lineage bias, where mutated HSCs preferentially contribute to the production of myeloid cells.

While detailed analyses of clone composition reveal that CHIP mutations originate in multipotent HSCs, the clonal progeny is predominantly concentrated within the myeloid compartment, specifically affecting monocytes and granulocytes, with a significantly lower mutational burden observed in lymphoid cells [29]. Accordingly, the most frequent mutations *DNMT3A* and *TET2* mutations, are most prevalent in myeloid cells for *TET2* and show a multilineage distribution for *DNMT3A*-carrying clones [30].

These consequences stem from the epigenetic roles of the involved genes. For instance, *DNMT3A* mutations cause focal DNA hypomethylation, which increases HSC self-renewal, while the loss of *TET2* leads to DNA hypermethylation and a hyper-inflammatory state. In essence, *DNMT3A* primarily enhances HSC self-renewal and clonal longevity, whereas *TET2* loss more characteristically drives a pro-inflammatory myeloid state, although these effects remain dependent on mutation specific characteristics. *TET2*-mutant myeloid cells exhibit altered cytokine production (notably IL-1ß) and inflammatory signaling, which has been directly linked to increased cardiovascular risk [6,31]. This skewed immune output ensures that the circulating pool of innate immune cells is enriched with mutated clones, which are primed for an altered inflammatory response [30].

The biological and clinical impact of CHIP is intrinsically tied to clone size, measured as the VAF. While the standard diagnostic threshold is often set at 2%, high-sensitivity sequencing can detect small clones at levels as low as 0.5%, reaching a prevalence of 39% in individuals older than 60 years [2,3]. This detection threshold is a critical methodological variable, as VAF levels may correlate with the intensity of systemic inflammatory output.

The prevalence of CHIP increases sharply with age, creating a significant biological intersection between individuals at risk for CHIP and late-onset AD [9,32]. Crucially, while CHIP has traditionally been viewed as deleterious and linked to systemic inflammation and cardiovascular disease, emerging data suggest mutation-specific effects that may uniquely modulate neurodegenerative pathogenesis [10,14].

## 4. Epidemiological Evidence Linking CHIP to Alzheimer’s Disease

Population-based studies have recently begun to directly examine the relationship between CHIP and the risk of AD, with the most comprehensive analysis to date provided by Bouzid et al. An overview of all epidemiological studies discussed in this section, including cohort characteristics and effect estimates, is provided in Table 1. Using longitudinal data from the Framingham Heart Study and the Cardiovascular Health Study, together with a case–control replication in the Alzheimer’s Disease Sequencing Project (ADSP), the authors demonstrate that CHIP carriers exhibit a reduced risk of AD dementia compared with non-carriers, independent of age, sex, and APOE genotype [10]. Meta-analysis across cohorts revealed a 36% reduction in AD risk among CHIP carriers [10].

A key methodological feature of this study was the consideration of clone size. While CHIP was detected at a standard ≥ 2% variant allele fraction (VAF) threshold in the community cohorts, analyses of the ADSP cohort were restricted to clones ≥ 8% VAF to harmonize clone-size distributions [10]. Protection from AD was primarily driven by larger clones (>8%), with weaker effects observed at lower VAF thresholds (>2%) [10]. Stratification by APOE genotype indicated that the protective association was strongest among APOE ε3/ε3 individuals, whereas no significant effect was detected in ε2 or ε4 carriers [10]. Neuropathological assessments in healthy individuals further demonstrated that CHIP carriers displayed lower Consortium to Establish a Registry for Alzheimer’s Disease (CERAD) scores, representing a reduced burden of neuritic amyloid plaque and reduced Braak staging. These factors indicate less advanced regional progression and severity of tau pathology [10]. Importantly, protection was observed across multiple CHIP-associated genes, suggesting a general effect of CH rather than a strictly gene-specific association [10].

Regarding protective associations, additional cohorts provide convergent evidence for protective associations. In the Women’s Health Initiative Memory Study, larger CHIP clones (≥8% VAF) were associated with a significantly reduced risk of clinically diagnosed probable dementia over a long-term follow-up of up to 25 years. By contrast, no association was observed for mild cognitive impairment [33]. Similarly, in the Chronic Renal Insufficiency Cohort, CHIP carriers exhibited a significantly lower risk of impairment when assessed for attention and executive function over a mean follow-up of approximately six years, suggesting domain-specific cognitive protection in patients with chronic kidney disease [19].

However, not all investigations support a uniformly protective role for CHIP. Yun et al., examining a small cohort of Korean patients with cognitive impairment using amyloid positron emission tomography imaging, reported no significant association between CHIP and brain β-amyloid deposition; however, this analysis was critically underpowered with only 58 participants [12]. More notably, Choi et al. reported an increased incidence of CHIP in AD cases compared with controls (*n* = 289), with effects driven specifically by APOE ε3/ε3 individuals [13]. In both their discovery cohort and validation cohort (namely ADSP), CHIP was associated with an increased risk of AD, providing evidence for a genotype-specific interaction that contrasts with the protective associations observed by Bouzid et al. Notably, both studies relied on the ADSP as a validation cohort yet arrived at strikingly opposed conclusions, underscoring the sensitivity of inferred CHIP effects to methodological heterogeneity. These differences likely stem in part from divergent sampling strategies and inherent selection bias in defining and selecting CHIP-positive individuals, including the stringent APOE-stratified sampling strategy employed by Bouzid et al., which focused the analysis on approximately 2400 individuals with the common *ApoE ε3* allele drawn from a cohort of more than 20,000 participants [10].

Broader neurodegenerative outcomes have also been examined in a large UK Biobank analysis. Liu et al. observed a modest increase in the risk of any neurodegenerative disease among CHIP carriers, with stronger associations for vascular neurodegenerative diseases and ALS; however, no significant association was detected for Alzheimer’s disease specifically [11] a finding likely constrained by the cohort’s younger demographic and the use of ICD-based diagnostic adjudication, which together lead to a significant underestimation of AD incidence and biomarker-confirmed cases.

Complementing this, Naito et al., leveraging whole-genome sequencing data from the ADSP cohort, reported on autosomal mosaic chromosomal alterations (mCA) in the blood. This is a distinct form of somatic clonal expansion involving large-scale structural events such as chromosomal gains, losses, and copy-neutral loss of heterozygosity. Such expansions were associated with increased risk of AD in an analysis of more than 24,000 individuals. Crucially, the study identified identical mCA clones within a subset of brain-resident microglia that exhibited a pro-inflammatory transcriptional profile characterized by the upregulation of MHC-II processing genes and markers of cellular senescence, providing direct evidence that large-scale somatic mosaicism in the blood can mirror pathological immune states in the CNS [34]. Collectively, these findings suggest that the impact of somatic mosaicism on the brain depends heavily on the specific genetic architecture of the clonal expansion.

Collectively, current epidemiological evidence indicates that the relationship between CHIP and Alzheimer’s disease is complex and highly context-dependent. Notably, three independent analytical frameworks have now been applied to the same ADSP cohort, yielding disparate results. This suggests that inferred CHIP-AD associations are strongly shaped by methodological choices, including CHIP definition (VAF 2% vs. 8%) and cohort stratification based on APOE genotype. While large CHIP clones in the ADSP cohort have been associated with a reduced AD risk and attenuated neuropathology [10], smaller clones, mosaic chromosomal alterations [34], and specific genetic contexts, most notably APOE stratification, may have increased susceptibility [13]. These discrepancies likely reflect differences in clone size thresholds, driver gene composition, the author’s APOE stratification approach, study design, and outcome definition, underscoring the need for harmonized CHIP detection criteria and prospective studies integrating genetic, neuropathological, and immune phenotyping to clarify the role of clonal hematopoiesis in AD pathogenesis. All published cohort analyses to date are summarized in Table 1.

## 5. Mechanistic Insights from Experimental and Translational Studies

**Figure 1:**
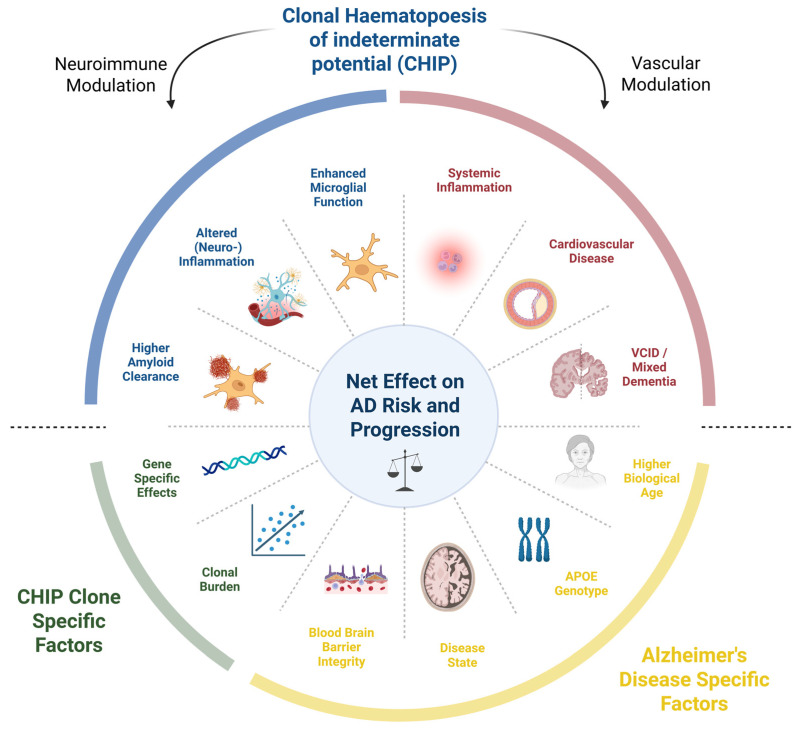
Conceptual model of clonal hematopoiesis as a bidirectional modifier of the risk and progression of Alzheimer’s disease. AD, Alzheimer’s disease; CHIP, clonal hematopoiesis of indeterminate potential; SVD, small vessel disease; VCID, vascular contributions to cognitive impairment and dementia (Created in BioRender. Kopp, K. (2026) https://BioRender.com/w7jvu15, accessed on 15 April 2026) [39].

To understand the complex interplay between CHIP-mutations and risk of AD, we propose a mechanistic model that accounts for the net effect of these diverse biological modifiers (Figure 1).

A central unresolved question linking clonal hematopoiesis to neurodegenerative disease is whether CHIP-carrying clones can directly influence the central nervous system by crossing the blood–brain barrier (BBB) and contributing to the brain’s immune compartment. This mechanism is supported by recent evidence identifying peripheral somatic clones within human brain-resident microglia [34] making it of relevance in AD, where aging, vascular dysfunction, and chronic neuroinflammation are known to compromise the integrity of the blood–brain barrier. This can lead to increased trafficking of peripheral immune cells as well as the above-mentioned microglial replacement of resident microglia by peripheral monocytes [24,36]. In this context, peripheral monocytes may infiltrate the brain and give rise to microglia-like cells, partially those that replace resident microglia under pathological conditions [37,38].

Most significantly, recent translational studies have established that CHIP-mutated clones infiltrate and populate the brain’s immune compartment. Post-mortem analyses by Bouzid et al. demonstrated that a subset of microglia from CHIP carriers harbored identical somatic mutations compared to those observed in peripheral blood, with mutation frequencies correlating with peripheral clonal burden [10]. This presence confirms that CHIP-derived myeloid cells can directly integrate into the central nervous system and may participate actively in neuroimmune processes relevant to AD pathogenesis [10,37].

### 5.1. Functional Consequences: Amyloid Clearance and Neuroinflammation

Experimental studies provide a mechanistic basis for the divergent effects of specific CHIP mutations in AD. In 5xFAD and APP/PS1 mouse models, *TET2*-deficient monocytes exhibit a fitness advantage, infiltrating the brain to differentiate into microglia-like cells (MLCs). These *TET2*-mutant MLCs exhibit a specialized phagocytic phenotype, characterized by the upregulation of lysosomal degradation pathways and the triggering receptor expressed on myeloid cells 2 (TREM2). These factors lead to a significant reduction in A*ß* plaque burden and disease progression [14].

At the molecular level, *TET2*-deficient immune cells exhibit altered inflammatory and chemotactic signaling, specifically the CXCL12/CXCR4 axis, which facilitates the migration of peripheral *TET2*-mutant myeloid cells toward sites of amyloid deposition [14]. Notably, this immune profile contrasts sharply with cardiovascular models, where *TET2*-deficient macrophages in the arterial wall adopt a hyper-inflammatory M1-like phenotype. In the context of atherosclerosis, these cells overproduce pro-inflammatory cytokines, including interleukin-1β and interleukin-6, via enhanced NLRP3 inflammasome activation, which accelerates plaque instability and vascular injury [15,40], highlighting the strongly context-dependent nature of CHIP-associated immune modulation. Ultimately, these findings reveal that the downstream consequences of CHIP differ substantially between vascular and neurodegenerative environments, where shared clonal variants may promote deleterious systemic inflammation while simultaneously enhancing protective amyloid clearance within the brain [30].

### 5.2. Gene-Specific CHIP Effects and APOE Interactions

Accumulating evidence indicates that the impact of clonal hematopoiesis on AD risk is strongly dependent on the identity of the driver gene and host genetic context [10,13]. While *TET2* mutations have been most consistently associated with enhanced amyloid clearance and attenuated neuropathology in experimental systems [10,14], the effects of *DNMT3A*- and *ASXL1*-mutant clones appear more heterogeneous. *DNMT3A*-mutant CHIP has shown protective associations with cognitive outcomes in some population-based and clinical cohorts as well as specific subgroups in these studies, particularly for larger clones and in select disease contexts [10,11,19,33]. However, it does not exhibit a comparable amyloid-clearing capacity in experimental AD models. Instead, it is strongly linked to pro-inflammatory and vascular phenotypes that may counterbalance neuroprotective effects in other settings [15].

The host’s genetic background further modifies these effects. Multiple epidemiological studies have identified the Apolipoprotein E genotype, particularly *APOE ε3*/*ε3* alleles, as a key modifier of CHIP-associated AD risk [10,13]. This interaction suggests that CHIP may exert its strongest influence in individuals lacking the dominant amyloid-promoting or amyloid-protective effects conferred by *APOE ε4* or *APOE ε2*, respectively [41]. Integrating the CHIP genotype with APOE stratification may, therefore, be essential for interpreting both protective and deleterious associations reported across cohorts [10,13].

### 5.3. Reconciling Experimental Findings with Epidemiological Heterogeneity

In summary, experimental and translational data reveal a remarkable functional versatility in CHIP-mutated clones. These findings suggest that the impact of somatic mutations on the aging brain is not fixed but is instead dynamically shaped by the specific driver gene, the local neuroinflammatory environment, and the integrity of the neurovascular unit [42]. Gene-specific effects, clonal burden, host genetic background, and neurovascular integrity all shape whether CHIP-derived immune cells exert protective or pathogenic influences in the aging brain [10,11,13,19,33,34]. These mechanistic insights provide a plausible biological framework for the heterogeneous and sometimes contradictory epidemiological findings observed across studies, particularly those derived from shared cohorts such as the ADSP [10,13,34].

## 6. Vascular Disease, Stroke, and VCID as Critical Confounders

A major challenge in interpreting associations between CHIP and AD arises from the strong and well-established link between CHIP and vascular pathology. CHIP robustly increases the risk of atherosclerotic cardiovascular disease, ischemic stroke, and cerebral small vessel disease, conditions that independently contribute to cognitive impairment and dementia [4,5,15,16]. These vascular effects are particularly relevant in aging populations, where mixed dementia phenotypes predominate, and vascular contributions to VCID frequently coexist with AD pathology [9].

Mechanistically, CHIP-associated mutations in genes such as *DNMT3A*, *TET2*, and *ASXL1* promote chronic inflammation, endothelial dysfunction, and accelerated atherogenesis through altered myeloid cell function and cytokine signaling [7,15,43] (Figure 1). In the cerebral circulation, these processes manifest as increased risk of ischemic stroke, white matter hyperintensities, lacunar infarcts, and microvascular injury, which are hallmarks of cerebral small vessel disease strongly associated with cognitive decline and executive dysfunction [16,17]. Importantly, vascular brain injury lowers cognitive reserve and amplifies the clinical expression of neurodegenerative pathology, complicating the distinction between AD and VCID [9].

From a clinical and pathological perspective, VCID encompasses a heterogeneous spectrum ranging from isolated vascular dementia to mixed forms in which vascular and Alzheimer-type pathologies coexist. Current consensus criteria emphasize that “pure” AD is relatively uncommon in advanced age, with most individuals exhibiting overlapping vascular, inflammatory, and neurodegenerative processes [9]. Within this framework, CHIP-related vascular injury may shift affected individuals toward a predominantly vascular or mixed dementia phenotype, even in the presence of substantial amyloid and tau pathology.

Most importantly, this framework provides a plausible explanation for the apparent protective associations between CHIP and AD reported in some epidemiological studies. First, competing risks play a decisive role: CHIP carriers experience increased cardiovascular morbidity and mortality, effectively limiting survival to ages at which clinical AD is most frequently diagnosed [4,7]. Second, diagnostic competition and misclassification may occur when vascular cognitive impairment dominates the clinical presentation, leading to a diagnosis of VCID or mixed dementia rather than AD. Third, survivor bias fundamentally enriches CHIP-positive cohorts for individuals with comparatively resilient vascular or immune phenotypes, particularly in long-term aging studies.

These considerations are consistent with emerging geroscience frameworks that conceptualize age-related diseases as interdependent manifestations of shared biological processes. Recent geroscience syntheses have identified clonal hematopoiesis as a systemic driver of inflammaging and vascular aging, with pleiotropic effects observed across organ systems, including the brain [7]. From this perspective, CHIP may simultaneously exacerbate vascular brain injury while modulating neuroimmune pathways relevant to amyloid handling, yielding net effects that vary according to genetic background, clonal burden, and competing pathologies.

Further support for this model can be derived from studies of other forms of somatic mosaicism. Mosaic chromosomal alterations, including loss of the Y chromosome (LOY), are strongly associated with cardiovascular disease, stroke, and dementia risk [34,44,45]. Recent whole-genome sequencing analyses demonstrate that autosomal mosaic chromosomal alterations are also associated with increased AD risk and are detectable in both blood and brain-resident microglia, implicating shared vascular and immune mechanisms [34,46]. Together, these findings suggest that distinct forms of somatic clonal expansion converge on vascular and inflammatory pathways that critically shape phenotypes of dementia.

In summary, the strong association between CHIP and vascular disease, stroke, and cerebral small vessel pathology represents a central confounder in studies on the risk of AD. Apparent neuroprotective associations may reflect competing vascular outcomes, diagnostic selection, or survival effects rather than true protection from Alzheimer-type pathology. Recognizing CHIP as a systemic modifier of vascular and brain aging, rather than a unidirectional risk or protective factor, is essential for reconciling discrepant findings and designing future studies that appropriately account for VCID and mixed dementia phenotypes.

## 7. Methodological Challenges and Sources of Contradictory Findings

The emerging literature linking CHIP to AD has produced heterogeneous and, at times, seemingly contradictory results. These discrepancies likely reflect methodological differences across studies rather than true biological inconsistency. Several key sources of bias and variability warrant careful consideration when interpreting existing findings and designing future investigations.

### 7.1. Detection Thresholds and Clonal Burden

One major source of heterogeneity arises from differences in CHIP detection thresholds and sequencing depth. Most large-population-based studies define CHIP using a VAF threshold of ≥2%, reflecting the lower limit of reliable detection in standard whole-exome sequencing pipelines [2,32]. However, several studies have demonstrated that clonal burden is a critical determinant of phenotypic effect. In particular, protective associations with AD and attenuated neuropathology were observed predominantly in individuals carrying large CHIP clones, typically defined by VAF thresholds of ≥8%; however, smaller clones showed weaker, null, or inconsistent associations [10,33].

To harmonize clonal burden across cohorts with differing sequencing platforms, the analyses by Bouzid et al. in the ADSP cohort restricted the CHIP-positive status to clones ≥8% VAF, underscoring the importance of clone size in interpreting disease associations [10]. In contrast, studies incorporating ultra-deep sequencing approaches that capture very small clones may identify biologically distinct populations, potentially diluting associations or introducing heterogeneity when clones with low VAF and uncertain functional relevance are included [13]. Crucially, these findings indicate that clonal burden may be a driver of disease association, suggesting that a threshold effect may exist where only larger clones possess the systemic impact necessary to influence neurodegenerative progression.

### 7.2. Tissue Specificity and Peripheral–Central Discordance

A second challenge relates to tissue specificity. CHIP is typically detected in peripheral blood, whereas AD pathology unfolds within the central nervous system. While recent evidence demonstrates that CHIP-associated mutations can be detected in microglia resident in the brain and correlate with peripheral clonal burden, this phenomenon appears to fluctuate and is context-dependent [10,34]. In contrast to earlier assumptions of restricted infiltration, it has been demonstrated that CHIP-mutated clones not only populate the brain’s immune compartment but can exhibit a higher variant allele fraction (VAF) within the isolated microglial pool than in the peripheral blood [10]. Specifically, single-nucleus sequencing has revealed that mutated microglia-like cells can constitute a substantial proportion of the brain’s immune niche, suggesting a selective fitness advantage or preferential recruitment of mutated myeloid cells to the central nervous system [10]. Further supporting this central accumulation, autosomal mCAs have recently been identified within brain-resident microglia, indicating that these somatic clones integrate into the neuroinflammatory landscape and potentially outcompete wild-type cells under pathological conditions [34]. Peripheral blood, therefore, provides an imperfect surrogate for clonal representation within the brain, as the circulating CHIP burden does not necessarily reflect the extent or functional relevance of CHIP-derived cells in the central nervous system. Differences in blood–brain barrier integrity, vascular pathology, and immune trafficking across cohorts may further modulate the extent to which peripheral CHIP influences central neuroimmune processes [26].

### 7.3. Survivor Bias and Competing Risks

Survivor bias and competing risks also act as significant confounders. As previously discussed, CHIP-associated cardiovascular mortality may prevent carriers from reaching the advanced ages at which AD is typically diagnosed [7,15]. This survival dynamic frequently generates spurious inverse associations in late-life cohorts, particularly when studies rely on clinical diagnoses rather than biomarker-based endpoints. Consequently, failing to account for these competing vascular outcomes often biases epidemiological estimates toward an appearance of neuroprotection.

### 7.4. Diagnostic Heterogeneity: AD Versus VCID

Diagnostic heterogeneity further complicates classification. The diagnosis of clinical Alzheimer’s disease often encompasses a spectrum of mixed pathologies, particularly in older adults, where VCID frequently coexists with amyloid and tau pathology [9]. Given the strong association of CHIP with cerebrovascular disease and small vessel pathology, CHIP carriers may be preferentially classified as having vascular or mixed dementia rather than classic AD. This diagnostic competition may obscure the underlying pathology of Alzheimer’s disease and contribute to divergent associations across studies using different diagnostic frameworks or outcome definitions for participant recruitment. Reliance on biological markers of amyloid and tau pathology, rather than clinical diagnosis alone, may help distinguish neurodegenerative from vascular contributions and clarify the relationship between CHIP and Alzheimer’s disease biology.

### 7.5. APOE Stratification and Genetic Context

Finally, inconsistencies in APOE stratification represent a critical methodological issue. Several studies report that the CHIP-associated risk of AD differs substantially by APOE genotype, with effects appearing most pronounced in individuals with APOE ε3/ε3 [10,13]. However, approaches to APOE adjustment and stratification vary widely, ranging from simple covariate adjustment to stringent genotype-restricted analyses that dramatically reduce sample size. Such differences can alter effect estimates, reduce statistical power, and produce discordant results, particularly in replication cohorts such as the ADSP.

Ultimately, these discrepancies underscore that APOE status is a fundamental biological modifier rather than a mere statistical confounder, making standardized genotype stratification essential for resolving conflicting findings in the field.

### 7.6. Summary of Methodological Challenges

Taken together, variation in detection thresholds, tissue specificity, survival dynamics, diagnostic classification, and genetic stratification provides a coherent explanation for the heterogeneous findings reported to date. Addressing these methodological challenges through harmonized CHIP definitions, explicit modeling of competing risks, standardized cognitive and biomarker outcomes, and integrative analyses incorporating vascular and neurodegenerative endpoints is essential for clarifying the true role of clonal hematopoiesis in AD and related dementias.

## 8. Proposed Risk Model: CHIP as a Bidirectional Modifier of AD Risk

Based on accumulating epidemiological and mechanistic evidence, we propose a revised risk model in which CHIP acts as a bidirectional, mutation and setting-dependent modifier of AD risk rather than a uniform pathological factor. Under this paradigm, certain CHIP mutations induce immune states that enhance phagocytic function, promote amyloid clearance, and attenuate maladaptive neuroinflammation. Conversely, other CHIP mutations, or identical mutations operating in different biological contexts, may exacerbate systemic inflammation, endothelial dysfunction, and cardiovascular pathology, indirectly increasing susceptibility to neurodegeneration through vascular injury and reduced cerebrovascular reserve [7,15]. These deleterious effects are particularly relevant in settings of high vascular burden, advanced age, or impaired blood–brain barrier integrity, where CHIP-associated vascular disease may dominate clinical outcomes and promote vascular or mixed dementia phenotypes. This approach reconciles the heterogeneous observations reported across studies and emphasizes the importance of stratified analyses.

The net effect of CHIP on Alzheimer’s disease risk is, therefore, determined by the balance between protective neuroimmune modulation and deleterious vascular and inflammatory influences, shaped by mutation type, clonal burden, disease stage, and host genetic background, including the APOE genotype [10,13]. Importantly, this model provides a coherent biological framework for reconciling seemingly contradictory findings across studies, including divergent results obtained from shared cohorts such as the ADSP.

By emphasizing stratified analyses that incorporate clonal burden, driver gene identity, vascular comorbidity, and biomarker-defined Alzheimer’s pathology, this bidirectional risk model moves beyond binary interpretations of CHIP as either protective or harmful. Instead, it positions CHIP as a systemic modifier of brain aging, the effects of which on AD risk emerge from the intersection of immune, vascular, and neurodegenerative processes (Figure 1).

## 9. Limitations and Knowledge Gaps

Despite growing interest, several fundamental knowledge gaps limit current understanding of how CHIP influences AD. Foremost, the causal relationship between CHIP and neurodegeneration remains unresolved, as existing evidence is largely observational. Whether CHIP directly modifies AD biology or primarily shapes clinical expression through vascular and immune pathways remains unclear.

The temporal dynamics of CHIP emergence in relation to AD pathogenesis are poorly defined. It remains unknown whether CHIP exerts its strongest effects during preclinical stages, influences disease progression, or modifies resilience to established pathology. Similarly, the extent to which peripheral clonal burden reflects functionally relevant clonal activity within the brain remains uncertain.

Addressing the current lack of biomarker integration is paramount, as few studies integrate longitudinal cognitive assessments with amyloid and tau biomarkers or neuropathological validation. Most large-scale cohorts (e.g., UK Biobank) rely on administrative health records, which often fail to distinguish between biological AD and mixed pathologies. In the absence of fluid or imaging biomarkers, it remains unclear whether the cognitive impairment observed in CHIP carriers results from accelerated amyloid-beta deposition or an increased burden of vascular frailty that effectively lowers the clinical threshold for dementia. Ultimately, without the granularity of biological staging provided by the A/T/N classification, it remains impossible to distinguish whether CHIP acts as a direct driver of primary proteinopathy or as a systemic modulator of the brain’s cognitive resilience to existing pathological burdens.

A fundamental deficiency in current research is the lack of longitudinal cohorts that integrate CHIP status with serial amyloid/tau PET imaging or CSF biomarker assays. Without these objective measures of Aß and tau burden, it remains impossible to determine whether CHIP-mutant clones actively influence the biological rate of proteinopathic accumulation or merely modify the clinical threshold for symptomatic expression. Finally, the way in which CHIP interacts with the host genetic background, particularly the APOE genotype, across biomarker-defined disease stages is essential for resolving the current heterogeneity in findings.

## 10. Conclusion and Clinical and Translational Implications: Toward a Hematology–Neurology Collaboration

If validated in prospective studies, CHIP could represent a novel biomarker for stratifying AD risk in aging populations. Given the high and steadily increasing prevalence of CHIP with age, incorporating CHIP status into AD risk prediction models has the potential to affect a substantial proportion of older individuals. Importantly, CHIP does not function as a binary risk factor but rather acts as a multidimensional modifier, where the magnitude of the clonal burden and gene-specific identification help predict clinical outcomes. Specifically, this includes accounting for mutation type, the affected protein domains, the total clonal burden, and the presence of vascular comorbidities or specific host genetic backgrounds.

From a clinical perspective, routine annotation of CHIP status in AD cohorts and longitudinal aging studies could enhance stratified analyses to identify participants most likely to benefit from anti-inflammatory or amyloid-clearing therapies. Integration of CHIP into biomarker-based diagnostic workflows, such as the amyloid/tau/neurodegeneration system, could help clinicians prioritize high-risk carriers for earlier PET imaging or more aggressive management of vascular comorbidities. Ultimately, these metrics help distinguish direct effects on AD biology from shifts in clinical diagnosis driven by vascular pathology. Such approaches could be particularly informative in individuals with mixed dementia phenotypes or discordant clinical and biomarker profiles.

Beyond risk stratification, CHIP raises translational questions regarding therapeutic targeting. While CHIP-associated immune alterations may be protective in some contexts, they are clearly pathogenic in others, particularly with respect to vascular disease. This duality suggests there is a need for functional immune assays alongside genomic profiling to discriminate between potentially beneficial and harmful clonal states. Addressing these challenges will require closer collaboration among hematology, neurology, and psychiatry to bridge expertise in clonal dynamics, immune function, and neurodegeneration.

## 11. Future Directions

Future research should prioritize nested case–control studies and Mendelian Randomization analyses within large, longitudinal cohorts that integrate deep genomic profiling of clonal hematopoiesis with repeated cognitive assessments, vascular imaging, and biomarker-based classification of AD while adding quantifiable endpoints such as longitudinal changes in plasma p-tau217 and neurofilament light chain. Multi-omics approaches incorporating transcriptomic, epigenetic, and immune phenotyping will be essential for resolving mutation-specific and stage-specific effects and linking peripheral clonal states to central nervous system pathology through spatial transcriptomics in post-mortem tissues.

On the experimental side, humanized and lineage-tracing models are crucial for differentiating causal mechanisms, including selective trafficking of CHIP-derived immune cells to the brain, interactions with resident microglia, and context-dependent effects on amyloid and tau pathology. Such models may help clarify why identical CHIP mutations confer divergent effects across vascular and neurodegenerative disease settings.

The potential interventional implications of CHIP also warrant careful consideration. Targeted innate immune modulatory strategy that reprograms microglial/macrophage function might modify CHIP-associated risk. However, indiscriminate suppression of immune activity could negate beneficial effects observed for certain mutations or clonal states. For instance, recent Phase 2 clinical evidence investigating TREM2-agonist antibody therapies has highlighted the complexities of myeloid reprogramming in neurodegeneration [47]. Although primary clinical endpoints were not met, these findings serve as a proof of point for the technical challenges involved in therapeutic immune modulation. Finally, ethical considerations surrounding CHIP screening, including disclosure, clinical actionability, and psychological impact, must be addressed, particularly as CHIP testing becomes more accessible.

Together, these efforts are essential for translating emerging insights into clinically meaningful strategies, while avoiding premature or overly simplistic interpretations of CHIP as uniformly protective or harmful.

## 12. Conclusions

CHIP has emerged as a significant yet complex biological feature of aging that intersects pathways relevant to AD. The collective evidence reviewed here indicates that rather than acting as a binary risk factor, CHIP serves as a systemic modifier of brain aging. Its net effect is dictated by the interplay between mutation-specific immune modulation and CHIP-driven vascular pathology. In some settings, particularly for large clones and specific mutations such as *TET2*, CHIP-associated immune alterations may enhance amyloid clearance and modulate neuroinflammation. In others, CHIP-driven vascular disease, stroke, and cerebral small vessel pathology may dominate, shifting the clinical presentation toward vascular or mixed dementia phenotypes and complicating diagnostic classification. Failure to account for these effects risks misinterpreting both protective and deleterious associations.

These insights underscore the necessity of moving beyond clinical labels to incorporate biomarker-verified AD pathology. This study also highlights the limitations of relying solely on clinical diagnoses without biomarker confirmation of Alzheimer’s pathology in aging populations.

Ultimately, advancing understanding of CHIP in AD requires multidisciplinary integration across hematology, neurology, psychiatry, geroscience, and vascular biology. Positioning CHIP as a context-dependent modifier provides a more coherent explanation of existing data than traditional binary models. Recognizing and accounting for these systemic influences will be essential for developing stratified, precision-oriented approaches required to accurately interpret AD biology in an aging population.

## Figures and Tables

**Table 1:** Summary of epidemiological studies linking clonal hematopoiesis to Alzheimer’s disease and cognitive outcomes [10,11,12,13,14,19,33,34,35].

Study	Cohort (*n* Individuals)	CHIP Definition	OR/HR (Outcome)	95% CI	*p*	APOE Effect	Outcome Summary	Strength	Weakness
**Protective Associations**									
**Bouzid et al.**	Framingham Heart Study (*n* = 2437) + Cardiovascular Health Study (*n* = 743) + Alzheimer’s Disease Sequencing Project (*n* = 2550; 1104 AD/1446 controls)	CHIP ≥ 8% VAF (ADSP); CHIP ≥ 2% VAF (community cohorts)	Meta OR 0.64 (AD dementia)	0.52–0.79	3.8 × 10^−5^	Protective in Apolipoprotein E ε3/ε3 only	Large-clone CHIP associated with reduced AD risk CHIP detected in the brain; associated with lower CERAD and Braak neuropathology	Multi-cohort; clone-size dependence; neuropathology validation	Retrospective sequencing cohort; heterogeneous CHIP definitions; effect mainly from large clones
**Matatall et al.**	United Kingdom Biobank (~500,000 adults) + AD mouse models (5xFAD and APP/PS1)	*TET2*-CHIP ≥ 2% VAF	OR 0.53 (AD risk)	0.32–0.86	0.01	*NA* *	Reduced AD risk; *TET2*-mutant myeloid cells infiltrate the central nervous system and enhance amyloid phagocytosis in mice	Human association plus mechanistic mouse data; causal pathway identified	Gene-specific; observational human data
**Jakubek et al.**	Women’s Health Initiative Memory Study (~5000 postmenopausal women)	CHIP ≥ 8% VAF	HR 0.62 (probable dementia)	0.41–0.94	0.025	*NA* *	Larger CHIP clones associated with reduced risk of adjudicated probable dementia; no association with mild cognitive impairment	Longitudinal follow-up; clinical dementia adjudication	Hazard ratio (not OR)
**Xiao et al.**	Chronic Renal Insufficiency Cohort (~500–1000 patients)	CHIP ≥ 2% VAF	HR 0.44 (attention); HR 0.60 (executive function)	0.26–0.76; 0.37–0.97	0.003; 0.04	*NA* *	CHIP carriers had significantly lower risk of attention and executive function impairment	Adjusted longitudinal cognitive testing	Disease-specific cohort; no dementia outcome
**Neutral/Inconsistent Associations**									
**Yun et al.**	Korean cognitive impairment cohort (*n* = 58)	CHIP ≥ 2% VAF	OR 0.85 (amyloid positivity)	0.13–7.29	0.686	Adjusted for Apolipoprotein E ε4	No association between CHIP and brain β-amyloid deposition	Imaging biomarker	Very small cohort; cross-sectional; underpowered
**Liu et al.**	United Kingdom Biobank (14,440 CHIP vs. 450,907 non-CHIP; total ~465,000)	CHIP ≥ 2% VAF	HR 1.10 (any neurodegenerative disease)	1.01–1.19	Not reported	*NA* *	CHIP associated with modestly increased risk of any neurodegenerative disease; AD specifically null	Large population cohort	Hazard ratios only; AD not primary outcome; not stratified on dementia subtypes
**Increased Neurodegenerative Risk**									
**Naito et al.**	Alzheimer’s Disease Sequencing Project whole-genome sequencing (*n* = 24,049)	Mosaic chromosomal alterations (not CHIP)	OR 1.27 (AD risk)	Not reported	1.3 × 10^−5^	Variable by Apolipoprotein E ε4	Mosaic chromosomal alterations associated with increased AD risk; same alterations were observed in brain microglia	Large sequencing cohort; brain validation	Preprint; limited stratification
**Choi et al.**	Discovery cohort (*n* = 289) + Alzheimer’s Disease Sequencing Project replication (~30,000)	CHIP ≥ 2% VAF	OR 2.89 (discovery); OR 1.32 (replication)	Replication: 1.05–1.67	<2 × 10^−7^; <0.02	Driven by Apolipoprotein E ε3/ε3	CHIP mutations are more frequent in AD vs. controls with increased AD risk in ε3/ε3 individuals	Deep sequencing; replication	Preprint; heterogeneous platforms
**Woo et al.**	Parkinson’s disease cohort (*n* = 341) + controls (*n* = 5003)	*TET2*-CHIP ≥ 1% VAF	OR 1.75 (Parkinson’s disease risk)	1.11–2.77	0.017	*NA* *	TET2-CHIP enriched in Parkinson’s disease; higher odds of Parkinson’s disease vs. controls	Gene-specific association; large control group	Cross-sectional

* NA: Not available; data were not reported in the original publications.

## Data Availability

No new data were created or analyzed in this study. Data sharing is not applicable to this article.

## Abbreviations

The following abbreviations are used in this manuscript:

AD	Alzheimer’s Disease
ADSP	Alzheimer’s Disease Sequencing Project
ApoE	Apolipoprotein E
A/T/N	Amyloid, Tau, and Neurodegeneration
BBB	Blood–Brain Barrier
CERAD	Consortium to Establish a Registry for Alzheimer’s Disease
CHIP	Clonal Hematopoiesis of Indeterminate Potential
CI	Confidence Interval
HSC	Hematopoietic Stem Cell
HR	Hazard Ratio
LOY	Loss of the Y Chromosome
mCA	Mosaic Chromosomal Alterations
MLCs	Microglia-Like Cells
OR	Odds Ratio
TREM2	Triggering Receptor Expressed on Myeloid cells 2
VAF	Variant Allele Fraction
VCID	Vascular Contributions to Cognitive Impairment and Dementia
